# Evaluation of a quality improvement intervention to prevent mother-to-child transmission of HIV (PMTCT) at Zambia defence force facilities

**DOI:** 10.1186/1472-6963-13-345

**Published:** 2013-09-08

**Authors:** Young Mi Kim, Maureen Chilila, Hildah Shasulwe, Joseph Banda, Webby Kanjipite, Supriya Sarkar, Eva Bazant, Cyndi Hiner, Maya Tholandi, Stephanie Reinhardt, Joyce Chongo Mulilo, Adrienne Kols

**Affiliations:** 1 Jhpiego/USA, an affiliate of Johns Hopkins University, 1615 Thames Street, Baltimore, MD 21231, USA; 2 Jhpiego/Zambia, an affiliate of Johns Hopkins University, Lusaka, Zambia; 3 Zambian Defence Forces, Defence Force Medical Services, Lusaka, Zambia

**Keywords:** Zambia, PMTCT, Antenatal care, Quality improvement, Task shifting

## Abstract

**Background:**

The Zambian Defence Force (ZDF) is working to improve the quality of services to prevent mother-to-child transmission of HIV (PMTCT) at its health facilities. This study evaluates the impact of an intervention that included provider training, supportive supervision, detailed performance standards, repeated assessments of service quality, and task shifting of group education to lay workers.

**Methods:**

Four ZDF facilities implementing the intervention were matched with four comparison sites. Assessors visited the sites before and after the intervention and completed checklists while observing 387 antenatal care (ANC) consultations and 41 group education sessions. A checklist was used to observe facilities’ infrastructure and support systems. Bivariate and multivariate analyses were conducted of findings on provider performance during consultations.

**Results:**

Among 137 women observed during their initial ANC visit, 52% came during the first 20 weeks of pregnancy, but 19% waited until the 28^th^ week or later. Overall scores for providers’ PMTCT skills rose from 58% at baseline to 73% at endline (p=0.003) at intervention sites, but remained stable at 52% at comparison sites. Especially large gains were seen at intervention sites in family planning counseling (34% to 75%, p=0.026), HIV testing during return visits (13% to 48%, p=0.034), and HIV/AIDS management during visits that did not include an HIV test (1% to 34%, p=0.004). Overall scores for providers’ ANC skills rose from 67% to 74% at intervention sites, but declined from 65% to 59% at comparison sites; neither change was significant in the multivariate analysis. Overall scores for group education rose from 87% to 91% at intervention sites and declined from 78% to 57% at comparison sites. The overall facility readiness score rose from 73% to 88% at intervention sites and from 75% to 82% at comparison sites.

**Conclusions:**

These findings are relevant to civilian as well as military health systems in Zambia because the two are closely coordinated. Lessons learned include: the ability of detailed performance standards to draw attention to and strengthen areas of weakness; the benefits of training lay workers to take over non-clinical PMTCT tasks; and the need to encourage pregnant women to seek ANC early.

## Background

Great strides have been made in the prevention of mother-to-child transmission of HIV (PMTCT), but much remains to be done. An estimated 390,000 children worldwide were newly infected with HIV in 2010, over 86% of them through mother-to-child transmission [1]. Mothers suffer as well: HIV has become a leading contributor to maternal mortality in sub-Saharan Africa [2], with an estimated 42,000 to 60,000 pregnant women worldwide dying because of HIV in 2009 [3]. UNAIDS has developed a Global Plan to virtually eliminate new HIV infections among children and sharply reduce the number of AIDS-related maternal deaths by 2015 [3]. The four-pronged approach involves:

• preventing HIV infection among women of childbearing age;

• meeting the needs of women living with HIV for family planning and birth spacing;

• ensuring pregnant women’s access to HIV testing and counseling and also to antiretrovirals (ARVs) to prevent HIV transmission from mothers to infants; and

• providing appropriate HIV care, treatment, and support for women and children living with HIV and their families.

Integrating PMTCT into maternal, newborn, child health, and reproductive health services has proven to be an effective strategy to reach HIV-infected mothers and their children [4,5]. In resource-limited settings, HIV counseling and testing conducted as part of routine antenatal care (ANC) has become the main gateway to HIV prevention, treatment, care, and support services for women and children [6].

Zambia—where the prevalence of HIV among women of childbearing age was 16% in 2007 [7]—has adopted this approach of integration to help reduce the spread of HIV. Like many countries with high HIV prevalence, Zambia has adopted a provider-initiated HIV testing and counseling model. New ANC clients are informed about PMTCT during group pre-test counseling. Unless a client opts out, providers then perform a rapid HIV test that produces results within one hour. Individual post-test counseling is offered as part of the standard package of ANC and delivery services [4]. This approach has been instrumental in increasing the uptake of HIV testing among pregnant women globally [6]. It has also contributed to high rates of HIV testing in Zambia, where 94% of pregnant women receive ANC services [7], and more than 95% of ANC clients accept the offer of HIV testing [8,9]. Among pregnant women who received ANC in 2010 and 2011, 98.9% were tested for HIV and received the results; 12.3% of them were found to have HIV [10]. The absolute number of HIV-positive women giving birth in Zambia has been slowly rising over the past decade and was estimated at about 97,000 in 2011 [10].

Identifying pregnant women who are infected with HIV is only the first step in PMTCT. The diagnosis must be followed by appropriate prophylaxis and treatment for mother and child. Zambian national guidelines follow recommendations issued by the World Health Organization (WHO) in 2010 [11]. These require testing pregnant women to determine whether their CD4 count is less than 350 mm^3^. If so, they are eligible for lifelong antiretroviral therapy (ART). If not, they are given a short course of ARV prophylaxis beginning in the second trimester, along with postpartum prophylaxis for the infant, to prevent vertical transmission. Zambia is planning to adopt new WHO guidelines issued in 2012 [12] that call for lifelong ART for all pregnant women with HIV, regardless of their viral load, but has not yet implemented them.

For many years, prophylaxis lagged behind HIV testing in Zambia. For example, a study of 60 primary health centers in rural and urban areas of Zambia in 2007–2008 found that only 17% of pregnant women diagnosed with HIV underwent CD4 screening, and there was further attrition before eligible women were given ART; lack of equipment to perform CD4 counts contributed to the low numbers [13]. The situation was better in the capital city of Lusaka, where 79% of pregnant women diagnosed with HIV underwent CD4 screening from 2007 to 2010 [8]. Nationwide, coverage has expanded rapidly in the past few years. In 2010, 80% of pregnant women with HIV in Zambia received some form of ARV prophylaxis to prevent transmission to the baby and 60.5% were assessed for ART eligibility through clinical staging or CD4 testing [10].

Improving the quality and the delivery of HIV prevention, care, and treatment services for women and children is an essential part of WHO’s PMTCT strategic vision 2010–2015 [5]. In Zambia, improvements in PMTCT service delivery have the potential to increase the number of women receiving ART and reduce the number of infants born with HIV [9,13,14].

These lessons are important for all providers of ANC services in Zambia, including the military-run health system. The Zambia Defence Force (ZDF) operates a network of 54 hospitals and clinics that serve military personnel, their families, and surrounding civilian communities; civilians make up 80% of all clients [15]. PMTCT is among the prevention strategies promoted by the ZDF’s HIV and AIDS Strategic Plan for 2009–2014.

The ZDF has been steadily expanding HIV-related services since 1993 [16], and more recently it has recognized the need to improve the quality as well as quantity of these services [17]. In 2006, the ZDF began introducing Jhpiego’s Standards-Based Management and Recognition (SBM-R®) approach at some facilities to improve the quality of HIV-related services. SBM-R uses a set of detailed standards to guide essential tasks performed by health care workers and to measure progress in service delivery [18]. A team of managers and health workers use SBM-R tools to identify and analyze weaknesses in service provision at their own facility and to develop an action plan to address them. Team members try to find solutions that rely on existing personnel and resources and that they can implement themselves, for example, coaching workers on specific tasks or posting job aids in consultation rooms. If necessary, however, the team may seek outside support for the action plan, for example, requesting additional staff or equipment from higher-level managers. In Zambia, the ZDF, with technical assistance from Jhpiego, provided training, supportive supervision, and on-site assistance to help staff teams analyze and address performance gaps and improve the quality of care.

Rawlins and colleagues found that the SBM-R approach improved the performance of reproductive, maternal, and child health services in Malawi [19]. However, there are no evaluations of the effectiveness of SBM-R interventions for ANC and PMTCT services, nor has there been an assessment of the suitability of the approach for military health systems. The purpose of this study is to evaluate the effectiveness of the SBM-R approach in improving the quality of PMTCT services at ZDF facilities.

## Methods

### Study design and sample

This study employed a quasi-experimental design that collected data at two points in time, pre- and post-intervention, from both intervention and comparison sites. Eight ZDF facilities participated in the study, including four intervention sites and four comparison sites. Sixteen ZDF facilities that had already implemented SBM-R were excluded from the study. Four of the remaining 38 facilities were purposively selected as intervention sites, based on their caseload and need for improvement in the quality of services. Four non-intervention sites were selected as comparison sites; they were matched as closely as possible to the intervention sites on ZDF branch, number of beds, and size of catchment population. The eight health facilities in the sample represent all three branches of the ZDF: they include two Zambian Air Force (ZAF) facilities, two Zambian National Service (ZNS) facilities, and four Zambian Army (ZA) facilities.

At each of the selected facilities, all health care providers responsible for delivering ANC/PMTCT services were asked to participate in the study and have their consultations with clients observed. The number of providers who were available to participate in the study during each round of data collection varied, depending on how many were away for training or meetings. A total of 39 providers participated in the baseline round of data collection and 48 providers in the endline round. The clients who participated in the study were a convenience sample of women seeking ANC services at the selected facilities; they included women making initial and follow-up ANC visits. The intention was to observe 25 ANC consultations at each facility during each round of data collection. However, this was not always possible if too few women came for ANC services while data collectors were present at the facility. A total of 387 ANC clients were observed; they included 93 clients at intervention sites and 98 clients at comparison sites during the baseline round of data collection and 86 clients at intervention sites and 100 clients at comparison sites during the endline round.

Group education sessions for pregnant women were also observed at each facility. The 41 group education sessions observed included 12 baseline and 9 endline sessions at the four intervention facilities, and 11 baseline and 9 endline sessions at the four comparison facilities.

### Description of the intervention

The SBM-R intervention began at the four study sites in September 2010. During an initial three-day visit to each site, ZDF and Jhpiego staff oriented facility managers to the quality improvement initiative and gave extensive training on the SBM-R assessment tools. They also introduced the tools and desired outcomes of the intervention to service providers. Working together with a facility team, ZDF and Jhpiego staff conducted a formal assessment of ANC services offered at the facility, using the SBM-R tools to identify strengths and weaknesses. Afterwards, they reviewed the results, analyzed root causes of some of the problems identified, and developed an action plan to address gaps in performance. The action plan guided providers on what they needed to do to improve service quality and solve problems. ZDF and Jhpiego staff also engaged in coaching and mentoring providers during this visit.

To support improvements in PMTCT services, ZDF and Jhpiego provided supplies and equipment where needed and conducted six days of onsite training for all providers offering PMTCT services. An additional six-day training course was held for PMTCT lay workers from the local community, 10 of whom were recruited at each facility as part of the intervention. The lay workers were trained to take over certain non-clinical responsibilities from service providers, notably conducting group education sessions for pregnant women seeking ANC services. At comparison facilities, Military Medical Assistants continued to lead group education sessions. Military Medical Assistants are recruited from the ranks of the ZDF and trained to provide basic health services; over the course of years, they may receive enough training to approach the skills of an enrolled nurse.

ZDF and Jhpiego followed up on these initial activities by making two- or three-day supportive supervision visits to each facility approximately twice a year. During these visits, supervisors observed consultations and mentored and coached providers to meet the standards set by SBM-R tools. At each visit, the facility team and project supervisors reviewed and revised the action plan for providers to implement in the coming months. Some problems could not be addressed by facility staff, and ZDF and Jhpiego offered assistance with these. For example, they communicated with the Defence Force Medical Services and donors about the lack of delivery rooms; eventually this led to the construction of maternity wards.

### Data collection

Baseline data were collected at all sites in August 2010. Post-intervention data were collected at the four comparison sites and two intervention sites from November to December 2011, prior to an administrative transition at the intervention sites. Data collection for the remaining two intervention sites occurred from March to April 2012, which allowed more time for the SBM-R process. The endline data collection employed the same tools as the baseline.

Data were collected from three sources: observations of ANC/PMTCT consultations, observations of group education sessions for pregnant women, and observations of the facility’s readiness to offer ANC/PMTCT services. The unit of analysis is the individual consultation, the group education session, and the facility, respectively.

#### Observation of consultations

Verbal consent was obtained from both the providers and clients prior to observing consultations. Before the consultation began, assessors asked clients background questions regarding their age, number of weeks pregnant, number of previous visits with this pregnancy, and number of prior pregnancies.

Assessors used an observation checklist based on the Zambia SBM-R assessment tools, which translate national guidelines into performance standards; each performance standard comprises a series of essential tasks known as verification criteria. Zambian guidelines for ANC and PMTCT are based on best practices set forth by the WHO [11]. During the ANC consultations, assessors recorded whether each verification criterion was observed, not observed or, on occasion, not applicable. The checklist included 12 performance standards related to ANC and PMTCT skills and 123 verification criteria (Table 1).

**Table 1:** Performance standards for ANC consultations

**Performance standard**	**Number of verification criteria**	**Content**
**PMTCT standards**		
PMTCT supplies and equipment	12	Preparation of drugs, supplies, and equipment needed for PMTCT
HIV counseling and testing	4	Pre- and post-test counseling, HIV testing
HIV/AIDS management in pregnancy	10	Counseling and referrals related to healthy lifestyles, ART, opportunistic infections, delivery, and continuity of care
Family planning counseling	4	Counseling on pregnancy spacing, breastfeeding, and contraception
*Overall PMTCT score*	*30*	
**ANC standards**		
ANC supplies and equipment	24	Preparation of drugs, supplies, and equipment needed for ANC
Interpersonal communication	7	Cordial and respectful treatment of woman
History taking	16	Personal information, obstetric and medical history, substance abuse, malaria prevention, test results
Physical exam	9	Privacy, hand hygiene, urine sample, vital signs, anemia, thyroid, edema, breast exam
Obstetric examination	10	Abdominal inspection, fetal heart rate and lie, external exam, hand hygiene and gloves, record-keeping
Individualized care	10	Lab tests, counseling and treatment on medical and lifestyle issues, birth planning
Follow-up	11	Addressing questions and concerns, reinforcing counseling, planning for return visit, record-keeping
Syphilis management	6	Treatment, counseling on HIV testing and condom use, return appointment
*Overall ANC score*	*93*	

#### Observations of group education sessions

During each round of data collection, assessors observed two or three group education sessions for pregnant women, on average, at each facility. Each session was led by a Military Medical Assistant at baseline and by a lay worker at endline, with the support of multiple counselors; no information was collected on how many different individuals were observed leading these sessions. Assessors asked the person leading each session for verbal permission to observe; the Institutional Review Board determined that it was not necessary to obtain consent from the women attending the sessions because of the public nature of the events. Assessors completed a checklist based on SBM-R assessment tools during each session, noting whether each of 28 verification criteria was observed or not. The four performance standards for group education covered the regularity of group education for pregnant women at the facility, the topics addressed, information checking, and the leader’s communication and education skills.

#### Facility readiness observations

Assessors completed a facility observation on the conditions needed to support quality ANC/PMTCT services at each site. The instrument was based on SBM-R assessment tools and covered 10 readiness standards, including drug requisitioning, storage, and availability, counseling by pharmacists, procedures, staffing, job descriptions, information and management systems, and client satisfaction. Assessors marked each of the 75 verification criteria as observed or not observed across the entire facility.

### Training of data collectors

To ensure that high-quality data were collected, assessors were recruited on the basis of their previous experience with field work. They also received special instruction on the purpose of the study, data collection tools, recruitment procedures, the consent process, data collection, and ethical issues. Two assessors were hired to collect baseline data, and four additional assessors were hired to collect endline data. All six assessors were midwives who had considerable experience with ANC/PMTCT services. In addition, all were third-party staff from the Ministry of Health who did not work at the ZDF facilities being assessed. ZDF personnel only assisted in helping the assessors gain admission to the military sites.

During each round of data collection, the assessors visited each of the participating facilities for three to four days. The goal was to complete the facility readiness observation instrument, to observe multiple group education sessions, and to observe at least 25 ANC consultations, although the latter was not always possible.

### Ethical considerations

This study was submitted to and approved by the University of Zambia Biomedical Research Ethics Committee and the Johns Hopkins Bloomberg School of Public Health Institutional Review Board. Observations and interviews were carried out in private, and informed consent was obtained from providers and clients.

### Data analysis

For each observed performance standard, the percentage of verification criteria achieved was calculated at baseline and at endline for each group (intervention or comparison group). We also calculated the percentage of verification criteria achieved for each facility readiness standard. An overall “percent achieved” score was calculated for provider performance, group education, and facility readiness.

In assessing provider performance, the performance standards for ANC consultations were analyzed separately for standards directly related to PMTCT and for other ANC standards not related to PMTCT; this highlights the effect of the intervention on PMTCT quality (Table 1). Four performance standards, including 30 verification criteria, contribute to the overall PMTCT score. Eight standards, including 93 verification criteria, contribute to the overall ANC score.

Next, a bivariate analysis was conducted of provider performance. We checked to see if the gains or declines were significantly different between the two groups. A *t*-test was used to detect whether any change from baseline to endline within each group was significant. (Small sample sizes did not permit this type of analysis for data collected on group education sessions or facility readiness).

Lastly, we conducted a multivariate analysis of provider performance. The outcome variable was the number of achieved or performed verification criteria. The model used was a generalized linear regression (glm) model with Poisson distribution and log link function, using an offset term of the total number of verification criteria. (The glm model with Poisson distribution was selected after comparing different models, including negative binomial regression, using the Akaike information criterion). The independent variables were the time point (baseline or endline), study group (intervention or comparison group), and the interaction of these two terms. Performing a multivariate analysis offered three advantages: First, the interaction term p-value would indicate if the change in one group was statistically different from the change in the other group. Second, the multivariate model controlled for ZDF branch (Army, Air Force, or National Service), holding that variable constant. Third, the multivariate models adjusted for clustering or correlation of responses within the same facility [20]. All analyses were performed using Stata 12.0 (College Station, TX).

## Results

### Provider and client characteristics

A total of 39 providers participated in the baseline round of the study, and 48 providers from the same eight facilities participated in the endline round. Most were nurses or nurse midwives (64.1% at baseline and 62.5% at endline). The remainder were Clinical Officers (25.6% at baseline and 16.7% at endline) and Military Medical Assistants (10.3% at baseline and 20.8% at endline). There was no significant difference between comparison and intervention sites in the distribution of provider types or in providers’ age, sex, or professional experience. On average, providers were 35.7 years old (SD=7.4) at baseline and 35.5 years old (SD=6.6) at endline. Over half were male (55.3% at baseline and 56.3% at endline). Participants had served as a ZDF health care provider for more than a decade on average (mean 10.2 years, SD=5.2, at baseline, and 10.2 years, SD=6.5, at endline).

Consultations with 191 ANC clients were observed during the baseline round and with 196 clients during the endline round. At baseline, the client’s mean age in the comparison group was slightly lower than in the intervention group (24.8 versus 26.3 years, p<.02); there was no difference between groups at endline (Table 2). Less than half of women were making their first ANC visit with this pregnancy, and only about one-fourth of women had no prior pregnancies. There was little difference between comparison and intervention groups, but the proportion of women making their first ANC visit fell from the baseline to the endline. A total of 83 women were observed during their first ANC visit at baseline and 54 at endline. Among these women, 52% came to the clinic during the first 20 weeks of pregnancy, while 19% waited until the 28^th^ week or later to seek ANC; there was no significant difference between comparison and endline groups.

**Table 2:** Characteristics of ANC/PMTCT clients observed, by round of data collection and study group

	**Baseline**			**Endline**		
**Characteristics**	**Comparison group**	**Intervention group**	**p-value**	**Comparison group**	**Intervention group**	**p-value**
**Age, in years**	(n=98)	(n=93)		(n=100)	(n=96)	
15–19	26.5	20.4	0.40	23.2	14.6	0.26
20–29	53.1	50.5		57.6	56.3	
30–39	17.4	26.9		18.2	28.1	
40+	3.1	2.2		1.0	1.0	
**No. of previous ANC visits with this pregnancy**	(n=98)	(n=93)		(n=100)	(n=96)	
0	42.9	45.6	0.76	27.8	32.3	0.58
1–2	23.5	25.6		39.2	35.4	
3+	33.7	28.9		33.0	32.3	
**No. of prior pregnancies**	(n=98)	(n=93)		(n=100)	(n=96)	
0	24.1	24.7	0.98	23.4	28.1	0.10
1	13.8	17.7		25.5	36.5	
2+	62.1	57.7		51.0	35.4	
**Among women making their first ANC visit, duration of pregnancy**	(n=42)	(n=41)		(n=24)	(n=30)	
≤ 20 weeks	40.5	61.0	0.17	50.0	53.3	0.50
21–27 weeks	40.5	24.4		20.8	30.0	
≥ 28 weeks	19.1	14.6		29.2	16.7	

Note: p-value from *χ*^2^ test.

### Provider performance on PMTCT

The overall PMTCT score at intervention sites rose by 16 percentage points, from 58% at baseline to 73% at endline (p=0.001). In contrast, comparison sites experienced a small and non-significant gain of less than one percentage point (Table 3). After the intervention, the overall PMTCT score was 21 percentage points higher at intervention than comparison sites (73% and 52%, respectively).

**Table 3:** PMTCT performance: results of bivariate and multivariate analyses of percentage of verification criteria achieved during ANC consultations, by data collection round and study condition

	**Bivariate analysis**				**Multivariate analysis** ^**a**^	
	**% achieved at:**		**Change from baseline to endline within group**		**Adjusted p-value for change within group**	**p-value for interaction**
**PMTCT performance standard and study condition**	**Baseline (n=191)**	**Endline (n=196)**	**% points**	**p-value**		
**PMTCT supplies and equipment**						
Comparison	67.8	62.8	-5.0	0.044	0.705	0.460
Intervention	76.8	85.0	+8.2	0.001	0.366	
**HIV counseling and testing**						
*During initial visits to ANC clinic*						
Comparison	88.5	77.1	-11.4	0.030	0.442	0.353
Intervention	75.8	87.1	+11.3	0.021	0.571	
*During return visits to ANC clinic*						
Comparison	20.6	35.4	+14.8	0.049	0.084	0.331
Intervention	12.5	48.3	+35.8	0.001	0.034	
**HIV/AIDS management in pregnancy**						
*During visits that do not include an HIV test*						
Comparison	3.2	21.6	+18.4	0.001	0.010	0.436
Intervention	0.7	34.1	+33.4	0.001	0.004	
*During visits that include an HIV test*						
Comparison	88.1	78.6	-9.5	0.040	0.288	0.385
Intervention	81.3	87.1	+5.8	0.083	0.745	
**Family planning counseling**						
Comparison	13.5	17.0	+3.5	0.418	0.687	0.477
Intervention	33.6	75.2	+41.6	0.001	0.026	
**Overall PMTCT score**						
Comparison	51.7	52.2	+0.5	0.875	0.961	0.241
Intervention	57.3	73.0	+15.7	0.001	0.003	

^a^ Results from generalized linear regression with Poisson distribution adjusted for ZDF branch.

Scores for the preparation of the drugs, supplies, and equipment needed for PMTCT rose by 8 percentage points at intervention facilities (p=0.001), but fell by 5 percentage points at comparison facilities (p=0.044).

**Figure 1:**
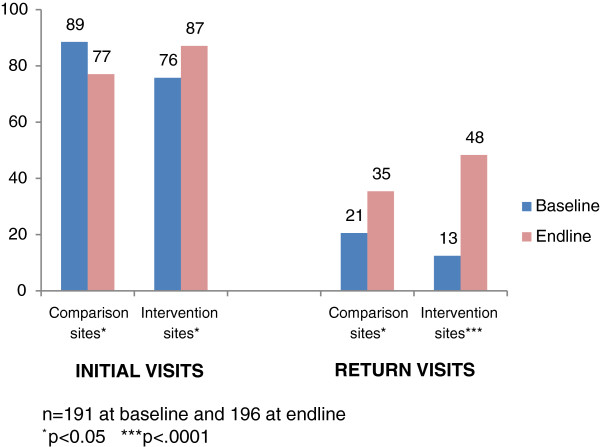
HIV counseling and testing: Percentage of four verification criteria achieved.

HIV testing typically takes place during the first visit a pregnant woman makes to the ANC clinic. When test results are negative, however, HIV tests are supposed to be repeated every three months during pregnancy and breastfeeding. Thus, scores for this performance standard are expected to be higher during initial than return ANC visits, as seen in the findings (Figure 1). Baseline scores during return ANC visits were so low, however, that they suggested providers were not consistently conducting the repeat HIV tests called for by PMTCT guidelines. Scores for women’s initial ANC visits increased by 11 percentage points from baseline to endline at intervention sites (p=0.021), but declined by the same amount at comparison sites (p=0.03) (Table 3). Gains were much greater during return ANC visits, with scores rising by 36 percentage points (p=0.001) at intervention facilities and 15 percentage points (p=0.049) at comparison facilities. The gains during return visits remained significant in the multivariate analysis for the intervention group (p=0.034), but not the comparison group.

**Figure 2:**
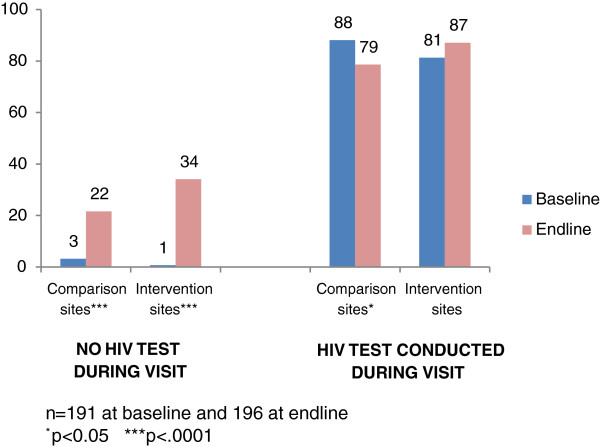
HIV/AIDS management in pregnancy: Percentage of 10 verification criteria achieved.

Providers are supposed to reinforce HIV counseling during every ANC visit, but findings suggest this was not happening prior to the intervention. Baseline scores for HIV/AIDS management in pregnancy were high during ANC visits that included an HIV test, but near zero during those that did not (Figure 2). Over the course of the intervention, scores increased significantly during ANC visits that did not include an HIV test, to a greater extent at intervention than comparison sites (33 percentage points, p=0.001, and 18 percentage points, p=0.001, respectively). The increases in both intervention and control groups remained significant in the multivariate analysis. This suggests that providers were increasingly likely to address HIV/AIDS throughout the course of a woman’s pregnancy. Changes in scores were smaller for visits that included an HIV test and did not retain their significance in the multivariate analysis. However, the trend was positive at intervention sites and negative at comparison sites.

Family planning to prevent unwanted pregnancies among HIV-positive women is a key element of the broader PMTCT strategy. The multivariate analysis showed significant improvement in family planning counseling at intervention sites, with the average score more than doubling from 34% at baseline to 75% at endline (p=0.026). There was little change in the comparison group over the same period (from 14% to 17%, ns) (Table 3). The interaction term in the multivariate analysis shows no significant difference between comparison and intervention sites in the amount and direction of change observed from baseline to endline, either for the overall PMTCT score or any specific performance standard.

### Provider performance on ANC

The overall ANC performance of providers improved by almost 7 percentage points in the intervention group (from 67% to 74%, p=0.001). It declined by about the same amount in the comparison group (from 65% to 59%, p=0.001). Neither change remained significant in the multivariate analysis.

According to the bivariate analysis, providers’ performance improved significantly on four of the eight ANC performance standards at intervention sites, including supplies and equipment, history taking, individualized care, and follow-up. Gains in the first three of these standards remained significant in the multivariate analysis (Table 4). In contrast, providers’ performance declined significantly on six of the eight standards at comparison sites. These included interpersonal communication, which also declined significantly at intervention sites. Scores were especially low for syphilis management in both groups and declined further over the study period. This was due to a shortage of reagents.

**Table 4:** ANC performance: results of bivariate and multivariate analyses of percentage of ANC verification criteria achieved during ANC consultations, by data collection round and study condition

	**Bivariate analysis**				**Multivariate analysis** ^**a**^	
	**% achieved at:**		**Change from baseline to endline within group**		**Adjusted p-value for change within group**	**p-value for interaction**
**ANC performance standard and study condition**	**Baseline (n=191)**	**Endline (n=196)**	**% points**	**p-value**		
**ANC supplies and equipment**						
Comparison	73.2	69.7	-3.5	0.004	0.626	0.135
Intervention	77.8	87.4	+9.6	0.001	0.001	
**Interpersonal communication**						
Comparison	98.4	86.7	-11.7	0.001	0.073	0.936
Intervention	97.7	85.1	-12.6	0.001	0.027	
**History taking**						
Comparison	48.1	51.1	+3.0	0.328	0.797	0.797
Intervention	50.0	56.7	+6.7	0.032	0.352	
**Physical exam**						
Comparison	72.5	59.4	-13.1	0.001	0.414	0.559
Intervention	82.6	79.1	-3.5	0.095	0.584	
**Obstetric examination**						
Comparison	60.0	49.5	-10.5	0.001	0.121	0.239
Intervention	54.8	54.9	+0.1	0.967	0.654	
**Individualized care**						
Comparison	58.9	52.6	-6.3	0.032	0.719	0.244
Intervention	60.8	79.7	+18.9	0.001	0.004	
**Follow**-**up**						
Comparison	80.3	55.7	-24.6	0.001	0.208	0.123
Intervention	79.0	86.1	+7.1	0.002	0.173	
**Syphilis management**						
Comparison	13.6	5.8	-7.8	0.124	0.010	0.114
Intervention	16.2	9.2	-7.0	0.203	0.234	
**Overall ANC score**						
Comparison	65.4	58.8	-6.6	0.001	0.583	0.307
Intervention	67.2	74.1	+6.9	0.001	0.084	

^a^ Results from generalized linear regression with Poisson distribution adjusted for ZDF branch and clustering within a facility.

The interaction term in the multivariate analysis shows no significant difference between comparison and intervention sites in the amount and direction of change observed from baseline to endline, either for the overall ANC score or any specific performance standard.

### Group education

Overall performance during group education sessions increased slightly at intervention sites, from 87% at baseline to 91% at endline (Table 5). There were dramatic gains in the two weakest areas at baseline: making sure that information is complete and correct by referring to job aids and checking with health workers (from 22% to 78%), and demonstrating good communication and education skills (74% to 94%). There was little change in the topics covered. At the endline, making sure that information was correct and complete remained the weakest area.

**Table 5:** Group education sessions: mean percentage of verification criteria achieved, by data collection round and study group

**Performance standards**^**a**^	**Comparison group**		**Intervention group**	
	**Baseline (n=11)**	**Endline (n=12)**	**Baseline (n=9)**	**Endline (n=9)**
Group education covers general ANC topics (4 criteria)	56.8	52.8	91.7	97.2
Group education covers HIV-related topics (11 criteria)	81.0	45.2	91.7	88.9
Leader makes sure information is complete and correct (2 criteria)	40.9	25.0	22.2	77.8
Leader employs good communication and education skills (11 criteria)	88.3	90.8	74.3	93.7
***Overall group education score (28 criteria)***	***77.5***	***56.5***	***87.1***	***91.1***

^a^ Missing values removed from numerator and denominator. N/A values recoded as missing.

At comparison sites, overall performance during group education sessions fell from 78% at baseline to 57% at endline. This reflects steep declines in the coverage of HIV-related topics (from 81% to 45%) and in making sure that information is correct and complete (from 41% to 25%).

### Facility readiness

Intervention facilities showed improvement on all but 1 of the 10 facility readiness standards listed in Table 6, and their overall readiness score rose from 73% at baseline to 88% at endline. Comparison facilities showed improvement on just 5 of the 10 standards, and their overall readiness score did not increase as much (from 75% to 82%).

**Table 6:** Facility readiness to offer ANC/PMTCT services: mean percentage of verification criteria observed at each facility, by data collection round and study group

**Facility readiness standards**^**a**^	**Comparison group (n=4)**		**Intervention group (n=4)**	
	**Baseline** ^**b**^	**Endline**	**Baseline**	**Endline**
Procedures for ordering, receiving, and issuing drugs are available (3 criteria)	100.0	100.0	83.3	100.0
ANC drugs are properly stored and managed (9 criteria)	85.1	88.2	86.1	91.7
Pharmacists offer ARV counseling (5 criteria)	60.0	100.0	75.0	100.0
Essential drugs for pregnant women are available (31 criteria )	88.2	87.8	74.3	91.1
Standardized procedures are used to provide essential package of integrated PMTCT services (10 criteria)	44.4	92.5	74.7	100.0
Sufficient staff are available for daily operations (3 criteria)	77.8	100.0	91.7	100.0
Job descriptions are available (1 criterion)	100.0	75.0	0	75.0
Information on morbidity and mortality is collected and used for decision-making (6 criteria)	30.0	29.2	62.5	29.2
Population-based calculations of PMTCT needs are used to set targets and assess performance (4 criteria)	100.0	68.8	68.8	93.8
Client satisfaction is assessed and analyzed (3 criteria)	33.3	16.7	41.7	50.0
***Overall readiness score (75 criteria)***	***74.6***	***82.0***	***73.1***	***87.5***

^a^ Missing values removed from numerator and denominator. N/A values recoded as missing.

^b^ Facility 5 is missing from baseline data.

Endline scores for most readiness standards were high at both intervention and comparison sites. However, intervention sites dramatically outscored comparison sites on two standards at the endline: using population-based calculations of PMTCT needs to set targets and assess performance (94% and 69%, respectively) and assessing client satisfaction (50% and 17%). Comparison and intervention sites scored equally poorly (29%) on using information on morbidity and mortality for decision-making.

## Discussion

### Improving PMTCT services

This study provides insight into the quality of ANC and PMTCT care provided at ZDF facilities. At intervention sites, providers’ general ANC skills were stronger than their PMTCT skills before the study began (67% and 58%, respectively). While their performance improved in both areas, gains for PMTCT were double those for ANC (16 percentage points and 7 percentage points, respectively), which closed the performance gap between them. Notably, provider performance at comparison sites remained stable for PMTCT and actually declined for ANC, suggesting that the SBM-R intervention was associated with improvements in provider performance at intervention sites.

While significant improvements were seen in every PMTCT skill area, family planning counseling improved the most, perhaps because it has become a priority for PMTCT services across Zambia. The provider training course devoted an entire module to family planning, and providers also received a family planning counseling kit containing job aids and a flip chart. On two standards—HIV testing and HIV management during pregnancy—performance improved more during return ANC visits than the initial visit. This is important because baseline data suggest that providers were not following PMTCT guidelines, which call for reinforcing counseling on HIV/AIDS throughout pregnancy and, if women are HIV-negative, repeating an HIV test every three months. Although the intervention markedly improved PMTCT services during return ANC visits, this remains an area of weakness and should be a focus of future quality improvement efforts.

It is not surprising that ANC performance showed less improvement than PMTCT performance at intervention sites since the intervention focused on PMTCT. However, certain ANC skills—preparation of supplies and equipment, history taking, individualized care, and follow-up—did improve significantly at intervention sites, while holding steady or declining at comparison sites. Providers at intervention facilities may have benefited from the mentoring and coaching that were integral to the supportive supervision visits conducted as part of SBM-R. Supervisors offered feedback and advice on all standards that were not met during observational visits, not just on PMTCT skills. The greatest improvement, a gain of 19 percentage points on individualized care, may also be due to the training providers received on family planning counseling; the course taught providers how to profile clients and tailor information to their needs, an approach similar to that taken in individualized care.

The interaction term in the multivariate analysis was not significant for the overall PMTCT and ANC scores or for any performance standards. Change over time between the two evaluation groups therefore was not statistically different. However, this may be due to low statistical power. A larger sample size may have been necessary to detect statistical interaction, but budget considerations, low ANC caseloads at ZDF facilities, and the fact that ANC services are not offered every day limited the number of observations that assessors could make.

The quality of care depends upon conditions at the facility as well as provider performance. Facility readiness scores were relatively high at baseline and continued to improve over the course of the study period at both intervention and comparison sites. In large part, this reflects the impact of a capacity-building initiative that has worked aggressively since 2007 to strengthen logistic management and the health supply system for ARVs, HIV tests, and laboratory commodities across all ZDF facilities, including both the intervention and comparison sites [21]. However, intervention sites made greater progress on facility readiness than comparison sites, probably because the SBM-R teams at intervention sites tended to focus their action plans on readiness and management issues. For example, they reorganized work schedules to reduce the impact of staff shortages, enforced drug requisition procedures, and analyzed and redesigned client flow. Notably, the intervention did not have a positive impact on using information on morbidity and mortality for decision-making. This is a weak area, suggesting the need for more attention to evidence-based medicine and specifically for refocusing the SBM-R facility assessment.

### Promoting early treatment

When women are infected with HIV, initiating ART earlier in their pregnancy has a positive impact both on maternal health and vertical transmission of the virus. Recent studies in sub-Saharan Africa have found that starting ART earlier in pregnancy suppresses a woman’s viral load to a lower level, reduces the risk of transmitting HIV to the infant during delivery and breastfeeding, and also reduces adverse pregnancy outcomes, including maternal mortality, stillbirth, and prematurity [22,23]. In Zambia, a retrospective cohort analysis found that the duration of ART prior to delivery was the single most important predictor of vertical HIV transmission: pregnant women who received ART for four weeks or less were five times more likely to transmit HIV to their infants than women on ART for at least 13 weeks [24]. The researchers concluded that pregnant women with HIV should start treatment 13 weeks before delivery to maximize the effectiveness of PMTCT. This means encouraging pregnant women to seek ANC no later than the second trimester, testing them for HIV as soon as possible, and ensuring prompt treatment or prophylaxis for those diagnosed with HIV.

Yet only about half of the women in this study who were making their initial ANC visit came early in their pregnancy, during the first 20 weeks; one-fifth waited until the 28^th^ week or later. This does not come as a surprise since an analysis of Demographic and Health Survey (DHS) data shows that women in sub-Saharan Africa tend to delay seeking ANC services longer than women in other regions [25]. According to the 2007 Zambia DHS, around half of pregnant women make their first ANC visit during the fourth or fifth month of pregnancy and one-fourth wait until the sixth month or later [7]. Prompting pregnant women to come earlier for ANC and HIV testing will require interventions that go beyond the facility and reach out to the community [26]. While research is limited, two studies suggest that training community volunteers to promote safe motherhood to women and their husbands can increase utilization of ANC. In Pakistan, female facilitators shared booklets and audiotapes on safe motherhood with support groups of women, while male facilitators shared the same materials with their husbands; a follow-up survey found that women in intervention clusters were three times more likely than other women to get ANC during the first two trimesters [27]. In Tanzania, safe motherhood promoters conducted home visits to encourage pregnant women and their husbands to book early for ANC and make birth plans; they also led educational meetings and video shows for the community. Over a two-year period, the proportion of women pregnant for the first time who booked an ANC appointment between 4 and 16 weeks tripled from 19% to 57% [28]. A similar approach could be effective in Zambia. PMTCT lay workers have already proven their worth in leading group education at ZDF facilities. They may be well-placed to promote early ANC visits in the communities surrounding ZDF facilities, through a combination of group meetings and home visits.

The findings suggest that most pregnant women are tested for HIV during their initial ANC visit. It is less clear whether they receive prompt treatment or prophylaxis, because this is not currently included in SBM-R performance standards. Adding a verification criterion that calls for offering ART to HIV-infected women as soon as possible, preferably during the same visit as the HIV test, would reinforce the need for prompt treatment. It could also be used to inform providers about the new guidelines for treatment that Zambia is planning to adopt, which are based on the Option B+ approach proposed by WHO [12]. These guidelines simplify service delivery and strengthen prevention by offering lifetime ART to all pregnant women with HIV, regardless of their viral load and without requiring a CD4 count. This will lessen delays in treatment by eliminating the need for CD4 testing, which is not available at all ZDF facilities.

### Task shifting

Like many sub-Saharan African countries, Zambia is dealing with a severe shortage of health care workers and has turned to task shifting, among other solutions, to meet the growing demand for HIV-related services [29,30]. This approach shifts tasks from more to less highly trained and qualified cadres, including to lay workers drawn from the community, as a way to expand the health work force and use it more efficiently [31]. Evidence from Zambia and other countries shows that, with sufficient training and supervision, lay workers can safely and effectively provide non-clinical services such as HIV education, are acceptable to health workers and patients, and can relieve the burden on overworked clinical staff [30,32-34].

Task shifting was an essential part of the intervention described here, as PMTCT lay workers were trained to lead group education sessions and to assist providers by completing forms, managing client records, and relaying HIV test results. Our findings confirm the ability of lay workers to offer good quality services, as group education scores increased dramatically at intervention sites after PMTCT lay workers took over the task from Military Medical Assistants. By the end of the study, group education scores at intervention facilities exceeded those at comparison sites by a wide margin (91% and 57%, respectively). Good design contributed to these results: the intervention met basic conditions for an effective community health worker program proposed by Hermann and colleagues, including the recruitment of workers from the local community, practically oriented training, clearly defined roles and standardized guidelines, and good supervision [35]. An earlier task-shifting initiative in Zambia, which also found that HV lay counselors outperformed health care workers, sheds further light on our findings [34]. Researchers concluded that the performance of lay workers benefited from specialized training and limited responsibilities. Health care workers, who have many clinical responsibilities and must juggle competing demands on their time, could not focus as intensely as lay workers on non-clinical tasks like education.

Comments made by providers and supervisors at SBM-R intervention sites indicate that PMTCT lay workers made a meaningful contribution to the facility’s workload. In their opinion, task shifting was one of the greatest benefits of the SBM-R intervention because it freed up more time for providers to spend with individual patients. While there is no quantitative data to confirm their impressions, other studies have noted a similar impact when lay workers take over time-consuming HIV counseling and education tasks [32,33].

The impact of PMTCT lay workers has been so positive that the ZDF is working to expand the initiative and the Ministry of Health is interested in copying it. However, past experience has identified serious challenges to the long-term sustainability of this kind of program. Lay workers need continuing training, supportive supervision, and performance review in order to maintain and improve their performance. They also need recognition, opportunities to learn new skills, and adequate compensation if they are to remain committed and stay on the job [31,32,35]. As a first step, the ZDF has begun offering PMTCT lay workers US$30 and a bicycle as incentives, but this may not be enough. Hermann and colleagues (2009) have argued that successful scaling-up of lay worker programs requires an appropriate regulatory framework, alignment with broader health system strengthening, and the flexibility to respond to changing needs and expectations [35].

### Study strengths and limitations

The study has two key strengths. First is the quasi-experimental design, with baseline and endline measures as well as intervention and comparison sites. This design protects against several threats to validity, including history effects (external events that may affect outcomes), maturation (natural improvements over time due to experience), and testing effects (earlier measurements affecting later measurements) [36]. Second, the study relied on direct observations of actual performance conducted by experienced health professionals without ties to the ZDF or Jhpiego, using detailed and comprehensive tools that reflect international best practices for low-resource settings and the Zambia service guidelines. This approach offers a more objective and reliable assessment of provider performance than interviews, self-reports, simulations, chart reviews, or role plays, which may be biased or reflect idealized situations [37].

However, interpretation of the findings is subject to certain limitations. Although ZDF facilities serve civilians as well as members of the military and their families, patients were not asked about their military or civilian status. Participating facilities and individuals were not randomly assigned to the intervention and comparison arms of the study, which is widely acknowledged as difficult to accomplish in practice [36]. Intervention facilities were deliberately selected for SBM-R because they were considered to be in greater need of quality improvement, so the facility sample may not be representative of all ZDF facilities. The results also are not generalizable to facilities that are not associated with the ZDF. The small sample size limited the power of the multivariate analysis to identify significant differences between intervention and comparison sites. Since providers try harder when under observation (the Hawthorne effect), observations likely overstate providers’ actual performance on the job. In addition, although inter-observer reliability was checked during training, it was not assessed during the study. Finally, the transfer of providers trained on SBM-R from intervention to comparison facilities may have narrowed differences between the intervention and comparison groups. In the long run, however, this will benefit the ZDF since providers tend to stay in the military.

## Conclusion

A quality improvement initiative that included multiple reinforcing activities—including provider training, supportive supervision, detailed performance standards, repeated assessments of service quality, facility action plans, and task shifting—succeeded in raising the quality of PMTCT services at ZDF facilities. Especially large gains were seen in family planning counseling, repeat HIV testing, and ongoing HIV/AIDS management throughout pregnancy. Group education also benefited.

SBM-R is designed to become an integral part of the management system at the facility level and to continue improving service quality over time, as staff teams repeat performance assessments and address the weaknesses that remain [18]. However, the short duration of this study does not shed light on the sustainability of the intervention and its continuing impact. Follow-up research is needed to assess whether performance gains are maintained over time, even as the extra training, supervision, and other inputs associated with the launch of the intervention are withdrawn and the facilities experience turnover in their staff.

While intervention took place in a military setting, the findings are also relevant to the civilian health system in Zambia, since the two are closely coordinated and share guidelines as well as many training and supervision activities. In fact, the Ministry of Health has begun to introduce SBM-R at its facilities, even as the ZDF continues to roll out the intervention at four more facilities each year. Key lessons learned include the ability of detailed performance standards to draw attention to and strengthen areas of weakness and the benefits of training lay workers to take over non-clinical PMTCT tasks. Future rounds of SBM-R could have an even greater impact by shortening the SBM-R tools so that they are more narrowly focused on PMTCT and can be used for provider self-assessments; ensuring that facility action plans address provider performance as well as facility readiness and management issues; and deploying lay workers in the community to encourage pregnant women to seek ANC earlier.

## Competing interests

The authors declare that they have no competing interests.

## Authors’ contributions

YMK, CH, and MT conceived of and participated in the design of the study. YMK was the principal investigator. WK oversaw the field work. SS and EB performed the statistical analysis. MC, HS, JB, SR, and JCM helped interpret the findings. AK and EB reviewed the literature and drafted the manuscript. All authors reviewed and helped revise draft versions of the manuscript and approved the final manuscript.

## Pre-publication history

The pre-publication history for this paper can be accessed here:

http://www.biomedcentral.com/1472-6963/13/345/prepub
